# Dr. Adams on Inflammation in Hydrophobia

**Published:** 1813-09

**Authors:** Joseph Adams


*To the Editors of the Medical and Physical Journal.*

GENTLEMEN,

THE very polite manner in which you inserted a compliment paid to my writings by a correspondent signing himself Ephebus, induces me to notice another paper contained in the same number. Mr. Aber, with a proper sense of justice, claims for Mr. Borrett a priority in suggesting a practice which has lately been much noticed: I mean the similarity between inflammation of the stomach and rabies canina. Without wishing to lessen the merit of that gentleman, give me leave to add, that a year before the date of his paper, in the last edition of Morbid Poisons, page 380, speaking of hydrophobia I have concluded thus: "In this surely it would be justifiable to push the *auxilium anceps* of Celsus much further than has hitherto been attempted, and not only the constant fatality of the disease, but the symptoms of a case of inflammation of the stomach, as described by Dr. Innes,* would sanction such an attempt.

After having turned to the paper to which Mr. Aber has directed your readers, (Med. and Phys. Journal, vol. xxi. p. 268) you must indulge me with a very few remarks. Without insisting on the turgescence of the vessels in the pia mater, the uncertainty of which I am ready to admit, it cannot be questioned that the appearances about the brain were those of inflammation. The affusion over the lobes, the firmness of the brain itself, and the fluid in the different cavities, are enough to ascertain this point. But the candour of Mr. Borrett will excuse me if I conceive the marks of inflammation about the stomach not so well ascertained. "The upper part of the œsophagus (says that gentleman, page 271) appeared free from inflammation, and covered with its fine cuticle till within four inches of the cardia, where there were evident marks of inflammation. The cuticular covering of the œsophagus was seen at this part as it were terminated by a loose frittered edge, and from this point to where the œsophagus expands into the cardiac orifice of the stomach, the cuticle was wholly gone. The œsophagus at the cardia, and for two inches upward, shewed strong marks of having undergone great inflammation. The cuticle was completely destroyed. The mucous membrane

* See Edinburgh Medical Essays abridged, vol. ii. p. 361. This patient, under the symptoms of hydrophobia, recovered after losing a hundred and sixteen ounces of blood.

had passed through the first stage of inflammation, had lost its redness and assumed an ash color, with dark-colored patches. The cardia had the same dark-colored spotted appearance, and the inflammation extended to nearly the breadth of the palm of the hand on the upper orifice and larger end of the stomach, which had a studded appearance of red points; these points becoming fainter as the inflammation extended in the direction of the larger curvature. In appearance the redness was not unlike a well-injected maternal part of the placenta. The rest of the stomach appeared free from inflammation."

I shall not pretend to urge that such a stomach had not been inflamed, but an attentive comparison of Mr. Borrett's accurate description with Mr. Hunter's account of the digestion of the stomach after death, may induce him to think with me that most of the appearances were rather to be ascribed to that cause than to inflammation. I shall only notice Mr. Borrett's happy allusion to a "well-injected maternal part of the placenta," and it may not be uninteresting to compare the appearances of the two as given by Mr. Hunter. "When we cut into the placenta," says that accurate pathologist, "its whole substance seems little less than a net-work, or spongy mass, through which the blood-vessels of the fœtus ramify; and indeed seems to be principally formed of the ramification of those vessels: it exhibits hardly any appearance of connecting membrane."*

In his paper on the digestion of the stomach after death, he describes two forms in which that phenomenon occurs; one in which the whole substance is eroded, and the contents of the stomach found loose in one of the cavities: this is uncommon. The other is so frequent that "there are few dead bodies in which *the stomach, at its great end*, is not in some degree digested; and one who is acquainted with dissections can easily trace the gradations." (Here Mr. Hunter, probably, would have explained himself, one who is in the habit of always examining those parts with a view to this particular appearance.) These gradations depend very much on the season of the year, and the mode of dying. If in the summer, the patient being in previous high health, and killed by violence soon after a meal, the substance of the stomach is sometimes completely eroded; but this is rare. If at any other period of the year, (and the progress to death has been rapid in all its stages)—"to be sensible of this effect

---

* Animal Economy, p. 167, 2d edition.

nothing nothing more is necessary than *to compare the inner surface of the great end of the stomach* with the other parts of its inner surface: (the latter or) the sound portions will appear soft, spongy, and granulated, and without distinct blood-vessels, opake and thick, while the others will appear smooth, thin, and more transparent; and *the vessels will be seen ramifying in its substance;*"* that is, by the digestion of the more spongy parts, the blood-vessels will be denudated, and even their finer extremities eroded, as appears by the rest of the paper, in consequence of which those vessels, from their transparency and number, will be all that occupy the sight, with here and there the studded appearance of red points so accurately marked by Mr. Borrett, and which, in the placenta, form the termination of the arteries.

We are much obliged to Mr. Borrett for a number of other cases he produces from different authors. These I shall notice in their order. Dr. Ferriar's cases so exactly correspond with Mr. Borrett's, that the same remarks may serve for each. Mr. Astley Cooper's cases appear to be inflammation of the stomach, but here we have no account of erosion, the patches of blood being effusions into the cellular substance, which cellular substance was destroyed by digestion in Mr. Borrett's and Dr. Ferriar's cases. Mr. Cooper's remarks on the dog's stomach, have been finely illustrated in Mr. Gilman's valuable publication. Dr. Beddoes's first case exhibited the fairest marks of inflammation about the pharynx, larynx, glottis, and neighbouring parts: his second case showed inflammation in the dura mater, the mucous membrane in the cervix of the bladder, and the villous coat of the ileum. In Dr. Powell's case there was at least as much appearance of inflammation in the head as in the stomach.

The mention of erosion in Dr. Pinckard's case, marks, as in the others, a digestion rather than inflammation of the surface. This is, in my opinion, confirmed by the description of the spotted and circumscribed redness below the cardia. All this will, I trust, be considered as matter of conjecture, which I am sure the candour of those gentlemen will receive only as offered to their further reflection.

After this, it may perhaps be expected that I should offer my own opinion, which I the more readily do, in hopes of being set right, or of receiving further information from the same gentlemen and your other correspondents.

---

* Animal Economy, p. 229, 2d edit.

† Med. and Phys. Journal, vol. xxiii.

That rabies is an inflammatory disease, most of the cases on record testify; but it appears as if the inflammation did not always fix on the corresponding parts of different subjects.

The valuable communication of Mr. Surr, contains the dissection of two, and the symptoms in four horses that died of this disease. The result of the whole is, that no inflammation of any consequence was found, excepting in the mucous membranes; and that different membranes were inflamed in different degrees in each. "In one the stomach had suffered inflammation even to gangrene; in the other no such appearance was found. The former of these horses neither ate nor drank during the symptoms, the latter did both: the œsophagus of the former was slightly inflamed, of the latter not at all. The appearance of the mucous membrane of the bladder in both was precisely the same. The urethra of each was inflamed. In one the whole mucous membrane, from the cribriform bone to the ends of the larger bronchial ramifications, was inflamed. In the other only five or six inches at the bronchial end, and scarcely entering the ramifications." After a few other remarks, Mr. Surr very judiciously observes, that the variety of symptoms, as well as of the seat of the diseased appearances, may teach us to account for some varieties of both, which have occurred in the human subject.

Mr. Gilman's publication furnishes us with some invaluable cases of his own, and judiciously selected from others. In many the stomach showed evident marks of inflammation, but this was neither constant nor uniform.

I shall mention only one other case, which, though not examined after death, strongly confirms Mr. Surr's opinion, that the seat of the disease is in the different mucous membranes. Mr. Bellamy is described by Mr. French as suffering an *unusual* titillation about the urethra, a contraction of the scrotum and penis to a degree of pain, and an emission of semen after making water, to which he had frequent calls. Dr. Fothergill confirms this account.* There can be no question in this instance that the urethra was inflamed, and probably the bladder.

Having now, I trust, brought forward a sufficient number of well-authenticated facts, the first remark I would make is, that in all subsequent examinations it is greatly to be desired that the inspections should not be confined to the head and stomach, or its communication with the mouth and neigh-

* Fothergill's Works, by Lettsom, vol. ii. p. 226 et seq.

bouring bouring parts. That by the various parts which have been found inflamed, it may at least be doubted whether these local actions are any thing more than sympathies, with some universal action excited in the constitution by the hydrophobic poison.

The appearances of local inflammation, it must be admitted, have in most cases been insufficient to produce death, as far as we may judge by analogy in other diseases. But it should be remarked, that the most fatal form of inflammation is that which continues increasing without any of the usual modes of termination. Thus in those unfortunate cases of inflamed throat, by which we have lost two valuable members of the faculty, no appearances of suppuration or gangrene were discovered. In croup we have sometimes an effusion of coagulated lymph; but this is neither necessary nor constant in the worst forms of the disease.

The next conjecture I would mention, and I hope all that I have said beyond the statement of facts will be considered as conjecture, is, that the hydrophobic poison may, like some other poisons, be determined in its influence to particular orders of parts, but not with the same certainty to the same parts in that order. Thus we find the variolous poison as well as the varicellous determined to the skin, and though usually with most violence to the face, yet this is not constant—the morbillous to the skin, and the membrane lining the bronchiæ—the poison of scarlatina to the throat and skin, sometimes both, and sometimes discoverable in only one. The venereal poison, when affecting the constitution, attacks occasionally the throat, the skin, and the bones; and we find a regularity in its progress to these different orders of parts. The atmospheric poisons have also their orders of parts. The deleterious air of hospitals, camps, and ships, besides the fever it induces, seems chiefly to exert its local influence on the brain; and in some cases where the effluvium has been particularly concentrated, death by apoplexy has almost superseded the accession of fever. The influenza produces its effect principally on the fauces and bronchiæ; and the autumnal impregnation of the atmosphere chiefly on the intestines, in the form of dysentery. The plague, however deleterious, shows its local effects principally in the glands.

In a review of the effects produced by these poisons, we have observed, that in some instances an affection is induced on several parts of the same order, in others only individual parts are affected, different in different subjects, but still confined to the same order; and we shall see that sometimes every part in that order may escape. Thus in the venereal disease the bones are sometimes found affected, usually the hardest, but it is uncertain which, or whether any. In the plague the glands, under the axilla, or in the groin, or in both, and even in the internal cavities: but sometimes the fatal issue supersedes every local affection.

In some of the worst cases, too, local affections occur apparently unconnected with the order of parts to which the poison is usually determined. Thus extensive ulcerations, like the carbuncle, are found in the plague, without any connection that we can discover with the glands; and in the early stages of the most inflammatory small-pox, we have inflammation of the pleura, or of the peritoneum, and always in a certain degree in the brain. The last may perhaps be considered in no other light than as symptomatic of some violent action excited in the constitution, and is probably always present in different degrees in every case of delirium.

It may be urged, that all I have said tends to throw greater uncertainty on the seat of hydrophobia. If this were really the case, it would be of less consequence should it lead to any certain diagnostic by which we may ascertain the proper mode of treatment, and act with as much decision and courage as the rapidity and usual fatality of the case demands. With this view I have endeavored to show that the variety of appearances in hydrophobia, described by different authors, is not greater than what is observed in other morbid poisons, the nature of which is better understood: that if these appearances cannot be all imputed to inflammation, many of them can arise from no other cause; and that there is nothing in any of them inconsistent with that process in the economy; consequently nothing which should make us fearful of early and copious bleeding.

I am, Gentlemen,

Your's, &c.

JOSEPH ADAMS.

COLLEC-

